# First They Came for Science

**DOI:** 10.34172/ijhpm.9035

**Published:** 2025-04-13

**Authors:** Mohammad Karamouzian

**Affiliations:** ^1^Centre on Drug Policy Evaluation, MAP Centre for Urban Health Solutions, St. Michael’s Hospital, Toronto, ON, Canada.; ^2^Dalla Lana School of Public Health, University of Toronto, Toronto, ON, Canada.; ^3^HIV/STI Surveillance Research Center, and WHO Collaborating Center for HIV Surveillance, Institute for Futures Studies in Health, Kerman University of Medical Sciences, Kerman, Iran.

## Dear Editor,

 Over the years, history has shown that political leaders who disregard science can have tangible, damaging impacts on population health. We see this in Hitler’s Germany, which refused important research because it was developed by “Jewish science,” or Stalin’s Soviet Union, which relied on Trofim Lysenko’s false agricultural theories and contributed to widespread famine and massive human casualties.^1,2^ These are not “ancient” examples; they remain relevant today, as more leaders target science and—often in tandem—marginalized populations for political gain. When credible evidence is ignored, it can serve as a pretext for discrimination, with communities experiencing marginalization bearing the brunt of both the denial of knowledge and the erosion of human rights. In the first few years of the HIV epidemic in the United States (US), the Ronald Reagan administration’s initial response was to dismiss the disease as a “gay plague.”^3^ This fostered an erroneous perception of the virus and delayed critical research funding and prevention campaigns, potentially costing countless lives. In South Africa, President Thabo Mbeki’s baseless skepticism about the link between HIV and AIDS prevented people living with HIV from accessing antiretroviral treatments, costing many lives.^4^ More recently, during the COVID-19 pandemic, we saw some of the world’s leading figures, including the US’s President Trump, downplay the severity of the virus and recommend ineffective treatments (eg, hydroxychloroquine), while undermining proven effective measures (eg, vaccines) advised by health experts.^5-7^ The resulting confusion weakened public trust in science.^7,8^

 Today, there is renewed concern over President Trump’s expanding set of executive orders, many of which undermine scientific progress, public health efforts, and human rights— and extend far beyond US borders. Examples include politicized budget cuts to programs essential for research and healthcare (eg, Medicaid, the Affordable Care Act, the National Institutes of Health, academic institutions, and the Centers for Disease Control and Prevention), aimed at curtailing or reallocating funding rather than reinforcing evidence-based initiatives. The administration also withdrew from the World Health Organization (WHO), canceled 83% of the US Agency for International Development (USAID) programs, and issued a stop-work order on the President’s Emergency Plan for AIDS Relief operations, weakening global health collaborations.^9,10^ Furthermore, rollback of nondiscrimination protections, such as redefining sex to exclude gender identity, further hinders inclusive research and healthcare for marginalized populations. Meanwhile, appointing individuals who question vaccine efficacy or disregard gender and racial health disparities erodes scientific integrity.^11^ Taken together, these actions may embolden other nations to adopt similar restrictive policies, further marginalizing at-risk populations and threatening both individual well-being and broader public health worldwide.

 Silence from the scientific community in the face of such attacks is not a neutral act; it is a form of complicity. From experience, we know that inaction allows harmful ideologies to fester. Martin Niemöller’s famous warning, “First they came for the socialists, and I did not speak out—because I was not a socialist... Then they came for me—and there was no one left to speak for me,”^12^ serves as a chilling reminder of the consequences of inaction and indifference. If we replace socialists with “scientists,” “LGBTQ+ communities,” “refugees,” or “racialized communities,” it becomes clear why academics must defend not only their disciplines but also the populations they serve. Indeed, the primary responsibility of the scientific community is to protect the public’s well-being and to advance knowledge. Staying silent in the face of disinformation and attacks on marginalized populations undermines this mandate, leading to preventable harm and eroding trust in science. Recent actions from Trump’s administration clearly show the urgent need to underscore how these actions affect population-level health; from reduced vaccination rates and a weakened infrastructure for future emergencies to restricted healthcare access for marginalized populations.^13^ If we fail to respond, these challenges will only intensify, deepening health inequities and threatening scientific progress.

 To counter these harmful actions, the scientific community should take a multi-faceted approach. First, we should support evidence-based policies by opposing rhetoric that contradicts peer-reviewed evidence, debunking disinformation in real time, and working with credible non-partisan journalists to make accurate, accessible science to the public. Second, we should engage and educate more than ever by partnering with media outlets, educators, community leaders, and faith-based organizations to promote scientific literacy and to disseminate clear, culturally-sensitive messages. Discrediting disinformation must remain central to public engagement, particularly for communities most affected by anti-science policies. Third, we should actively engage at every level of government—federal, state, and local—by speaking up at legislative hearings, serving on advisory committees, and shaping regulatory policies to ensure evidence-based decision-making. Policy-makers rely on credible data to inform public health policies, and when the federal government’s stance conflicts with scientific consensus, local or state actions can still protect marginalized communities. For example, healthcare practitioners in states supportive of transgender rights can expand relevant training and services, even amid restrictive federal policies. By using federalism strategically, the scientific community can preserve spaces where evidence-based practices thrive and push back against disinformation. Fourth, we should protect people experiencing marginalization at all costs. The effect of such attacks on specific marginalized groups (eg, the LGBTQ+ or racialized communities), have far-reaching public health repercussions, from reduced preventive HIV care to worsening mental health outcomes. Scientists have an ethical and professional duty to collaborate with grassroots organizations and legal advocates to ensure these populations retain access to essential healthcare and services. Fifth, we should ensure that institutions are held accountable. Academic institutions and professional societies should challenge leaders and officials who endorse discriminatory or anti-science policies, ensuring that harmful rhetoric does not go unchecked. Lastly, philanthropic organizations and major research think tanks, both within and beyond the US, should strengthen scientific infrastructure, particularly where universities have shown a disappointing lack of response. These institutions also need to bolster scientists’ ties to professional societies, including international ones, by maintaining or expanding professional development support and international collaborations. Such measures provide “shelters” for critical research and protect the integrity of evidence-based science, even in politically challenging environments.

 Rejecting science and targeting marginalized groups are not isolated actions; they are part of a broader erosion of societal values and equity. Such actions are not taking place in a vacuum; they are part of a wider agenda to de-legitimize evidence-based science, smear marginalized populations and dismiss opposition as merely ideological rather than factual. However, remaining silent in the face of such actions allows dangerous ideologies to spread unchecked, remain unchallenged, and gain momentum to the point where they jeopardize public health, equity, and scientific advancement. The scientific community cannot remain neutral, but has to act in defense of evidence and in solidarity with people experiencing oppression. Niemöller’s warning is still relevant today, an injury to one is a threat to all. If evidence is suppressed and people experiencing marginalization are targeted, entire societies will feel the adverse health and well-being consequences. At this critical time when harmful policies from this administration seek to undo decades of progress in global public health, we need to speak up and reclaim our commitment not only to scientific integrity, but to justice, equity, and human dignity.

## Ethical issues

 Not applicable.

## Conflicts of interest

 Author declares that he has no conflicts of interest.
